# Catalyzing urea hydrolysis using two-step microbial-induced carbonate precipitation for copper immobilization: Perspective of pH regulation

**DOI:** 10.3389/fmicb.2022.1001464

**Published:** 2022-09-16

**Authors:** Zhong-Fei Xue, Wen-Chieh Cheng, Lin Wang, Yi-Xin Xie

**Affiliations:** ^1^School of Civil Engineering, Xi’an University of Architecture and Technology, Xi’an, China; ^2^Shaanxi Key Laboratory of Geotechnical and Underground Space Engineering (XAUAT), Xi’an, China

**Keywords:** microbial-induced carbonate precipitation, ureolytic bacteria, copper metal, two-step biomineralization, copper-ammonia complex

## Abstract

Microbial induced carbonate precipitation (MICP) has recently applied to immobilize heavy metals toward preventing their threats to public health and sustainable development of surrounding environments. However, for copper metallurgy activities higher copper ion concentrations cause the ureolytic bacteria to lose their activity, leading to some difficulty in forming carbonate precipitation for copper immobilization (referred to also as “biomineralization”). A series test tube experiments were conducted in the present work to investigate the effects of bacterial inoculation and pH conditions on the copper immobilization efficiency. The numerical simulations mainly aimed to compare with the experimental results to verify its applicability. The copper immobilization efficiency was attained through azurite precipitation under pH in a 4–6 range, while due to Cu^2+^ migration and diffusion, it reduced to zero under pH below 4. In case pH fell within a 7–9 range, the immobilization efficiency was attained via malachite precipitation. The copper-ammonia complexes formation reduced the immobilization efficiency to zero. The reductions were attributed either to the low degree of urea hydrolysis or to inappropriate pH conditions. The findings shed light on the necessity of securing the urease activity and modifying pH conditions using the two-step biomineralization approach while applying the MICP technology to remedy copper-rich water bodies.

## Introduction

Copper (Cu) is an indispensable trace element for human health, plant and animal growth, and it has an activating effect on some key enzymes in cellular metabolism (Facchin et al., 2013). However, it can impose serious threats to organisms if the concentration exceeds the legal limit (Elalfy et al., 2021; Guo et al., 2021). Most of the copper in nature exists as compounds (i.e., copper minerals), and in China, the development of copper mining, smelting, and processing has raised the potential of their migration and diffusion in surrounding environments (Bai et al., 2021b,c; Hu et al., 2021a,2022a,b; Wang et al., 2022b; Xue et al., 2021; Yu et al., 2021). Nowadays there are various physical and chemical measures available for remedying copper-rich water bodies. However, these methods are time-consuming, costly, and not environmental-friendly (Qdais and Moussa, 2004; Wei et al., 2005; Fu and Wang, 2011; You et al., 2018; Chen et al., 2022a,b; Li et al., 2022; Wang et al., 2022a). In recent years, the microbial-induced carbonate precipitation (MICP) technology has attracted extensive attention as an alternative to traditional measures (Achal et al., 2012a; Chen and Achal, 2019; Ye et al., 2021; Xue et al., 2022a,b).

The MICP technology can precipitate carbonates between soil particles and has been widely applied to calcareous sand reinforcement (Jiang et al., 2019; Lai et al., 2020; Rahman et al., 2020; Zhang et al., 2020; Cui et al., 2021; Xiao et al., 2021; Yang et al., 2022), while studies on the remediation of heavy metals using the MICP technology are markedly limited (Kang et al., 2015; Li et al., 2021; Wang et al., 2022e,f). The principle of the MICP technology is to catalyze urea hydrolysis through secreting the urease using the ureolytic bacteria, discharging hydroxide and ammonium ions (see Eqs. 1–3) and subsequently yielding carbonates. Heavy metal ions and calcium ions could co-precipitate with bacteria as nucleation sites in the biomineralization process (see Eqs. 4–7) (Li et al., 2013). As a result, the “net” effect of such a reaction corresponds to an increase in surrounding pH (Schwantes-Cezario et al., 2017; Cui et al., 2022). The use of carbonates aims to capsulize heavy metal ions by forming carbonate precipitation (termed immobilization of heavy metals hereafter), preventing their migration and diffusion (Achal et al., 2011; Achal et al., 2012b; Jiang and Soga, 2019; Chen and Achal, 2019; Schwantes-Cezario et al., 2020; Liu et al., 2021; Mujah et al., 2021; Zeng et al., 2021). Ali et al. (2022) reported that strain *B. diminuta* isolated from soil could immobilize Cd^2+^ and Zn^2+^ in solution by co-precipitation. In addition, extracellular polymers (EPS) secreted by bacteria can provide nucleation sites and promote bacteria to immobilize heavy metal ions in solution (Chen et al., 2016; Casas et al., 2020; Qiao et al., 2021; Kim et al., 2021).


(1) $${\text{CO(NH}}_{2}{)}_{2}+{\text{H}}_{2}\text{O}\to {\text{2NH}}_{3}+{\text{CO}}_{2}$$

(2) $$2{\text{NH}}_{3}+2{\text{H}}_{2}\text{O}↔{\text{2NH}}_{4}{}^{+}+2{\text{OH}}^{-}$$

(3) $${\text{CO}}_{2}+2{\text{OH}}^{-}↔{\text{HCO}}_{3}{}^{-}+{\text{OH}}^{-}↔{\text{CO}}_{3}{}^{2-}+{\text{H}}_{2}\text{O}$$

(4) $${\text{Ca}}^{2+}+\text{Cell}\to \text{Cell}-{\text{Ca}}^{2+}$$

(5) $${\text{M}}^{2+}+\text{Cell}\to \text{Cell}-{\text{M}}^{2+}$$

(6) $$\text{Cell}-{\text{Ca}}^{2+}+{\text{CO}}_{3}{}^{2-}\to \text{Cell}-{\text{CaCO}}_{3}\left(\text{s}\right)$$

(7) $$\text{Cell}-{\text{M}}^{2+}+{\text{CO}}_{3}{}^{2-}\to \text{Cell}-{\text{MCO}}_{3}\left(\text{s}\right)$$

Recent studies indicated that the copper immobilization efficiency generally maintains at low levels compared to other heavy metals (Achal et al., 2011; Li et al., 2013; Mugwar and Harbottle, 2016; Bai et al., 2019, 2021a; Hu et al., 2021b; Wang et al., 2022c,d). It is due to the fact that copper ions bind to the functional groups of the urease, and as a result, its spatial structure is badly modified causing its denaturation and inactivation (Krajewska, 2008; Seplveda et al., 2021). Among all the positive divalent heavy metals, copper has the highest toxicity on the urease activity except mercury, and securing the urease activity against copper metal is deemed a challenging task while introducing the MICP technology (Zaborska et al., 2004; Jiang et al., 2019). Increasing the initial urea concentration may improve the resistance of the urease against copper metal. However, the higher the urea concentration, the higher the surrounding pH, and the higher the potential of forming complexes unfavorable for the immobilization of copper metal (Ferris et al., 2004; Torres-Aravena et al., 2018; Duarte-Nass et al., 2020; Liu et al., 2020; Seplveda et al., 2021; Tarach et al., 2021; Chen et al., 2022a,c; Xue et al., 2022c).

Conducting a closer look to the literature on the biomineralization, however, reveals a number of gaps and shortcomings. To this end, the present work proposes a two-step biomineralization approach; the first step allows the ureolytic bacteria hydrolyze urea to discharge the amount of carbonate and hydroxyl ions necessary for forming carbonates in the second step. The second step mainly aims to use the ureolytic bacteria as nucleation sites to precipitate carbonate, capsulizing copper metal (Torres-Aravena et al., 2018; Duarte-Nass et al., 2020). Prior to the second step where the urease is inoculated to the medium containing copper metal, the amount of carbonate and ammonia ions necessary for forming carbonates in the second step is already yielded in the first step, mitigating the effect of Cu^2+^ toxicity. In addition, different inoculation proportions may consider in the second step to modify the surrounding pH, thus preventing the formation of complexes unfavorable for securing the copper immobilization efficiency. The objectives of this study are: (1) To conduct test tube experiments to investigate the effects of bacterial inoculation and pH surrounding conditions, (2) to highlight the necessity of modifying pH and distinguish the speciation of precipitation against different pH ranges using the numerical simulations, and (3) to propose the two-step biomineralization approach to secure the immobilization efficiency.

## Materials and methods

### Ureolytic bacteria culture

*Sporosarcina pasteurii*, a basophilic ureolytic bacterium, was used in the present work. It was activated in a sterile liquid medium, which consists of 20 g/L yeast extract, 10 g/L NH_4_Cl, 20 g/L urea, 10 mg/L MnSO_4_⋅H_2_O, 24 mg/L NiCl_2_⋅6H_2_O. The surrounding pH for the sterile liquid medium was adjusted to 8.8 using 1 M solution of NaOH. The activated ureolytic bacteria were mixed with glycerol using a ratio of 7:3 and stored at –20 °C. They were subjected to shaking culture at 30 °C and 180 rpm for 30 h. Further, the chemicals of urea, MnSO_4_⋅H_2_O, NiCl_2_⋅6H_2_O, NaOH, and Cu(NO_3_)_2_⋅3H_2_O were diluted to given concentrations, respectively, and applied to the subsequent test tube experiments.

### Urease activity measurement

The urease activity (termed UA hereafter) under different bacterial inoculation proportions was measured in Cu^2+^ contained 0.5 M urea solution, which aims to evaluate the effect of Cu^2+^ toxicity on the ureolytic bacteria and urease activity. The concentration of Cu^2+^ and the bacterial inoculation proportion were 5, 10, and 20 mM, and 1:9, 1:3, and 1:1, respectively.

pH, EC (electric conductivity), and UA, while catalyzing urea hydrolysis, were measured using a benchtop pH meter (Hanna Instruments Inc. HI2003) and a benchtop conductivity meter (Hanna Instruments Inc. HI2314), respectively. UA was measured on a basis of the ureolysis rate, as recommended by Whiffin et al. (2007); 2 mL final reaction solution is mixed with 18 mL 1.11 M urea, and EC is measured at 0 min and 5 min after the mixing. UA can be evaluated using the equation below:


(8) UA=EC5-EC05×10×1.11(mMUreamin)-1

where *EC*_0_ and *EC*_5_ are electrical conductivity at 0 and 5 min, respectively. NH_4_^+^ concentration of the final reaction solution is measured at 0, 24 and 48 h, respectively, and the method for measuring NH_4_^+^ concentration corresponds to the modified Nessler method (Whiffin et al., 2007). There were three replicates for each test set.

### Numerical simulations

To evaluate the effect of Cu^2+^ concentration and pH on the speciation of precipitation and copper immobilization efficiency, the biomineralization process was reproduced using the Visual MINTEQ software package, although the process of urea hydrolysis has been neglected. The initial concentration for NH_4_^+^ and CO_3_^2–^ was calculated in accordance with the bacterial inoculation proportion. Table 1 summarize the parameters applied to the numerical simulations.

**TABLE 1:** Parameters applied to the numerical simulations of the copper immobilization efficiency against Cu(NO_3_)_2_ concentration and pH considering bacterial inoculation proportions of 1:9, 1:3, 1:1, and 3:1, respectively.

Bacterial inoculation proportion	Ion concentration (mM)					pH
		
	Cu^2+^	NO_3_^–^	NH_4_^+^	CO_3_^2–^	Cl^–^	
1:9	5, 20, 40, 60, 80	10, 40, 80, 120, 160	85.3	33.3	18.7	0–13
1:3			213.25	83.25	46.75	
1:1			426.5	166.5	93.5	
3:1			639.75	249.75	140.25	

### Test tube experiments

This part aims to elaborate more about the details applied to the test tube experiments. First, a Cu(NO_3_)_2_ solution at concentrations varying in a 0–60 mM range was prepared, while the ureolytic bacteria were cultivated with yeast extract and ammonia nitrogen, during which time urea at 333 mM was also added to the culture medium. Second, the urea hydrolysis proceeded with the bacterial inoculation proportions of 1:9, 1:3, and 1:1, respectively, for 48 consecutive hours. The aforesaid two-step biomineralization approach is the first proposed by the authors and primarily aims not only to discharge NH_4_^+^ and OH^–^ preventing not only the effect of Cu^2+^ toxicity but the formation of complexes unfavorable for securing the copper immobilization efficiency. NH_4_^+^ and Cu^2+^ concentrations were measured at 0, 24, and 48 h, respectively. An atomic spectrophotometer (Beijing Purkinje General Instrument TAS-990) was responsible for the Cu^2+^ concentration measurements. The copper immobilization efficiency can be evaluated as follows:’


(9) $$\mathrm{Immobilization}\,\mathrm{efficiency}=\left(\left(C_{0}-C_{1}\right)/C_{0}\right)\times 100\%$$

where *C*_0_ and *C*_1_ are Cu^2+^ ions concentration before and after remediation, respectively. Table 2 summarize the scheme applied to the test tube experiment. There were three replicates for each test set.

**TABLE 2:** Scheme applied to the test tube experiments.

Parameters applied to the first step	Parameters applied to the second step	Concentration of Cu(NO_3_)_2_ (mM)
Urea at 333 mM NH_4_Cl at 187 mM	1:9	20, 40, 60
	1:3	20, 40, 60
	1:1	0–50

## Results and discussion

### Test tube experiments

#### Effect of bacterial inoculation

UA is an important indicator that determines the growth and reproduction of the ureolytic bacteria during the biomineralization process. Furthermore, the higher the UA, the higher the resistance of the ureolytic bacteria against copper metal (Song et al., 2017). Considering copper metal can significantly impede the bacteria’s growth and reproduction (Zaborska et al., 2004), the degradation mechanism is summarized as copper metal binding to the functional groups of the urease and modifying its spatial structure, thus causing its denaturation and inactivation (Krajewska, 2008). Duarte-Nass et al. (2020) suggested that increasing the initial urea concentration could improve the resistance of ureolytic bacteria against Cu^2+^ toxicity. Despite that, such high initial urea concentration, however, turns the surrounding pH into alkaline environments promoting the formation of copper-ammonia complexes unfavorable for securing the copper immobilization efficiency (Liu et al., 2018; Duarte-Nass et al., 2020).

**FIGURE 1:**
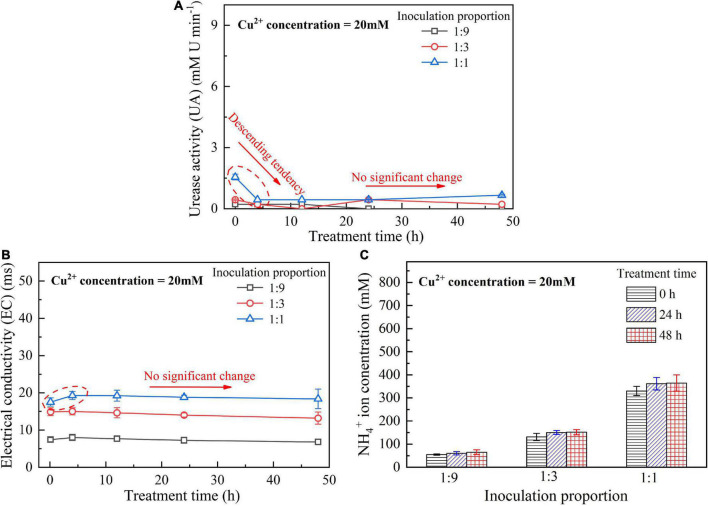
**(A)** UA vs. treatment time relationship, **(B)** EC vs. treatment time relationship, and **(C)** NH_4_^+^ vs. treatment time relationship when subjected to Cu^2+^ concentration at 20 mM.

**FIGURE 2:**
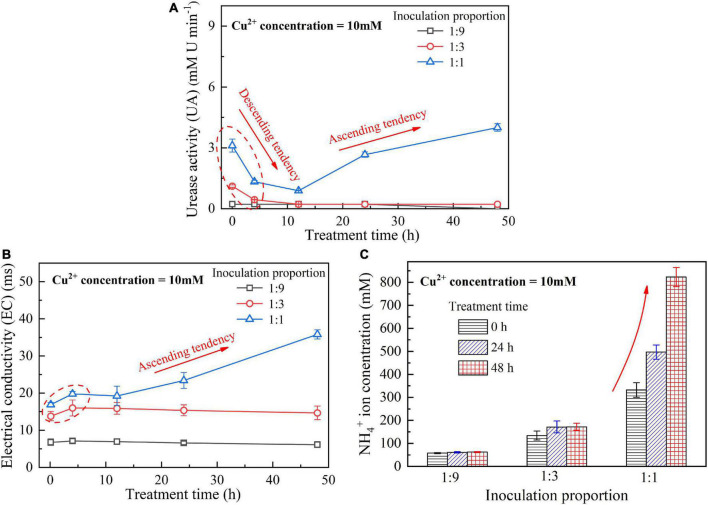
**(A)** UA vs. treatment time relationship, **(B)** EC vs. treatment time relationship, and **(C)** NH_4_^+^ vs. treatment time relationship when subjected to Cu^2+^ concentration at 10 mM.

**FIGURE 3:**
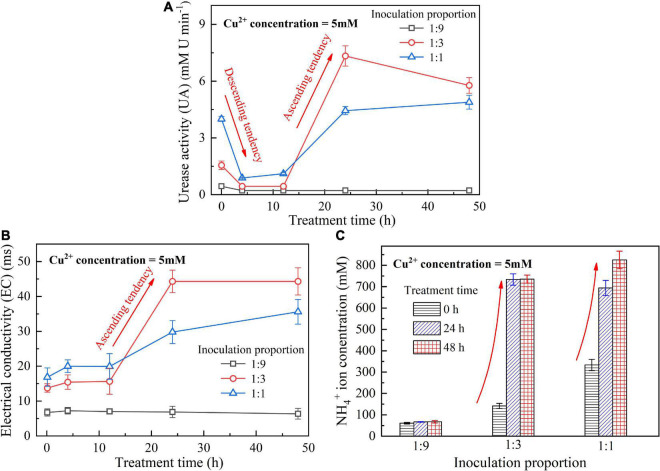
**(A)** UA vs. treatment time relationship, **(B)** EC vs. treatment time relationship, and **(C)** NH_4_^+^ vs. treatment time relationship when subjected to Cu^2+^ concentration at 5 mM.

When subjected to 20 mM Cu^2+^, UA goes into a decline for the bacterial inoculation proportion = 1:1, while for the bacterial inoculation proportions = 1:3 and 1:9, it shows a smaller change (see Figure 1A). UA for all the bacterial inoculation proportions reduces to approximately zero 4 h after the beginning of bacterial inoculation, indicating that the effect of Cu^2+^ toxicity depresses the growth and reproduction of the ureolytic bacteria and causes some difficulty in catalyzing urea hydrolysis. For this reason, the measurements of EC and NH_4_^+^ show a small change because of a small number of NH_4_^+^ and OH^–^ discharged, as depicted in Figures 1B,C. UA for the bacterial inoculation proportion = 1:1 goes into a decline at the very beginning of bacterial inoculation when subjected to 10 mM Cu^2+^, and goes up 12 h following the commencement of bacterial inoculation, as shown in Figure 2A. In contrast, UA for the other two inoculation proportions presents a negligible change all long. These results indicate that the ureolytic bacteria for the bacterial inoculation proportion = 1:1 could remain its activity when subjected to 10 mM Cu^2+^, and for the other two inoculation proportions, the effect of Cu^2+^ toxicity depresses the growth and reproduction of the ureolytic bacteria reducing the secretion of the urease. The measurements of EC and NH_4_^+^ provide testimony of the above argument, as shown in Figures 2B,C. Given the inoculation proportion = 1:1, UA going up 12 h after the beginning of bacterial inoculation is attributed to the reduction in the effect of Cu^2+^ toxicity, induced by the formation of copper-ammonia complexes. In other words, the formation of copper-ammonia complexes causes an inability of depressing the growth of the ureolytic bacteria and enhances the resistance of the urease against Cu^2+^ toxicity. When subjected to 5 mM Cu^2+^, the behavior can also be recognized as UA going down rapidly and going up till the end of the process and becomes more distinct compared to 10 mM Cu^2+^. UA for the inoculation proportion = 1:3 even surpasses that for the inoculation proportion = 1:1 12 h after the commencement of bacterial inoculation. The relatively higher urea concentration for the inoculation proportion = 1:3 may be considered as the main cause leading to such phenomenon. EC and NH_4_^+^ measurements give testimony to the argument made, as shown in Figures 3B,C.

On the whole, when subjected to 20 mM Cu^2+^, either for higher inoculation proportions or lower inoculation proportions the majority of the ureolytic bacteria lose their activity in the first 4 h because of the effect of Cu^2+^ toxicity. For this reason, a small number of NH_4_^+^ and OH^–^ are discharged and EC, therefore, shows a small increase. The effect of Cu^2+^ toxicity badly depresses the growth and reproduction of the ureolytic bacteria for the subsequent 8 h. The ureolytic bacteria that remain active 12 h after the commencement of bacterial inoculation begin showing their resistance against Cu^2+^ toxicity. The formation of copper-ammonia complexes reduces the effect of Cu^2+^ toxicity on the ureolytic bacteria and UA. This phenomenon becomes more pronounced when subjected to lower Cu^2+^ concentrations and higher bacterial inoculation proportions (e.g., 1:3 and 1:1). When subjected to 10 mM Cu^2+^, the ureolytic bacteria remain active only when the bacterial inoculation proportion is raised to 1:1, whereas the ureolytic bacteria, when subjected to 5 mM Cu^2+^, remain active even when the inoculation proportion is reduced to 1:3. These results lead us to summarize that although higher inoculation proportions can improve the resistance of the ureolytic bacteria against Cu^2+^ toxicity and promote the secretion of the urease, their use is accompanied by discharging more OH^–^ throughout the biomineralization process, turning surrounding environments into alkaline conditions and promoting the copper-ammonia complexes formation. The copper-ammonia complexes largely raise the potential of Cu^2+^ migration and diffusion, causing an inability of securing the copper immobilization efficiency. As indicated by Figure 3C, an improvement in EC and NH_4_^+^ may cause misleading interferences concerning the use of high inoculation proportion of 1:1 for improving the copper immobilization efficiency. Therefore, it is argued that higher inoculation proportions pave the way to secure copper immobilization efficiency. In addition to UA, particular attention to the surrounding pH conditions should also be given, preventing a reduction in the copper immobilization efficiency by the copper-ammonia complexes formation.

#### Effect of surrounding pH conditions

**FIGURE 4:**
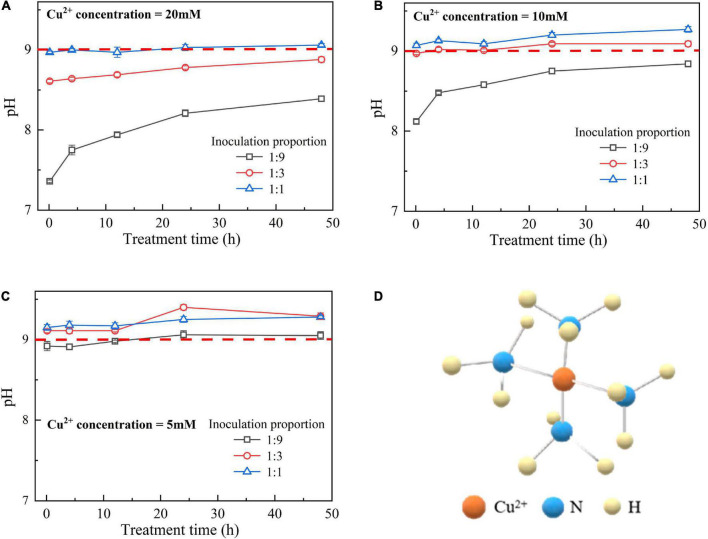
**(A)** pH vs. treatment time relationship under Cu^2+^ concentration at 20 mM, **(B)** pH vs. treatment time relationship under Cu^2+^ concentration at 10 mM, **(C)** pH vs. treatment time relationship under Cu^2+^ concentration at 5 mM, and **(D)** schematical illustration of copper-ammonia complex.

The temporal relationships of pH against the bacterial inoculation proportion = 1:9, 1:3, and 1:1 when subjected to 20, 10, and 5 mM Cu^2+^ are shown in Figures 4A–C, respectively. The results from the previous section indicate that when subjected to 10 mM Cu^2+^, the ureolytic bacteria remaining active is present only when the inoculation proportion is raised to 1:1, while the bacteria that remain active, when subjected to 5 mM Cu^2+^, presents even when the inoculation proportion is as low as 1:3. The value of surrounding pH corresponding to the these results, however, exceeds 9 (Duarte-Nass et al., 2020). As discussed, the ureolytic bacteria can be characterized as UA going down in the first 4 h after the commencement of bacterial inoculation, and UA going up since after 12 h (see Figures 2A, 3A). The bacteria that remain active 12 h after the commencement of bacterial inoculation can reproduce and catalyze urea hydrolysis, discharging NH_4_^+^ and OH^–^. This is deemed as the main cause leading to the value of pH in excess of 9.

**FIGURE 5:**
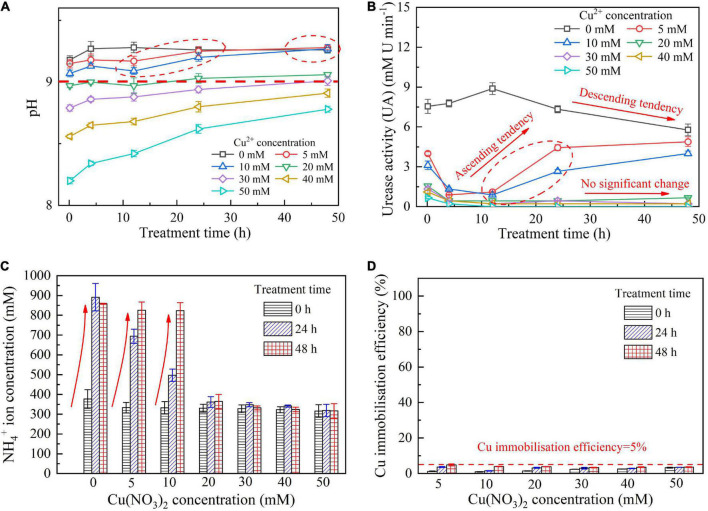
**(A)** pH vs. treatment time relationship, **(B)** UA vs. treatment time relationship, **(C)** NH_4_^+^ vs. Cu(NO_3_)_2_ concentration relationship and, **(D)** copper immobilization efficiency vs. Cu(NO_3_)_2_ concentration relationship for the bacterial inoculation proportion = 1:1.

pH below 9 is attained using 20–50 mM Cu^2+^ where the effect of Cu^2+^ toxicity can depress the growth and reproduction of the ureolytic bacteria (see Figures 5A,B). The ureolytic bacteria remain active when subjected to 0–10 mM Cu^2+^, discharging NH_4_^+^ throughout the biomineralization process (see Figure 5C). It is worth to note that the copper immobilization efficiency could be as low as 5% under the inoculation proportion being 1:1, and such low copper immobilization efficiency still holds true when Cu^2+^ concentration decreases to 5 mM (see Figure 5D). These results conflict with our consensus that lower Cu^2+^ concentrations can ease the effect of Cu^2+^ toxicity on the ureolytic bacteria and promote the secretion of the urease by the ureolytic bacteria, thus improving the degree of urea hydrolysis and subsequently the copper immobilization efficiency. There are two underlying mechanisms revealed by the present work. Although the ureolytic bacteria remain active when subjected to 0–10 mM Cu^2+^, the highest inoculation proportion of 1:1 not only eases the effect of Cu^2+^ toxicity on the ureolytic bacteria and UA but turns the surrounding pH into alkaline conditions (pH > 9), promoting the formation of copper-ammonia complexes. The copper-ammonia complexes raise the potential of Cu^2+^ migration and diffusion and reduce the copper immobilization efficiency to as low as 5%. Furthermore, despite pH below 9 and no copper-ammonia complex formation, the effect of Cu^2+^ toxicity badly depresses the ureolytic bacteria and UA when subjected to a range of 20–50 mM Cu^2+^, reducing the degree of urea hydrolysis. The lower the degree of urea hydrolysis, the lesser the carbonate precipitated, and the lower the copper immobilization efficiency. The reduction in the degree of urea hydrolysis reduces the copper immobilization efficiency to approximately 5%.

### Numerical simulations

**FIGURE 6:**
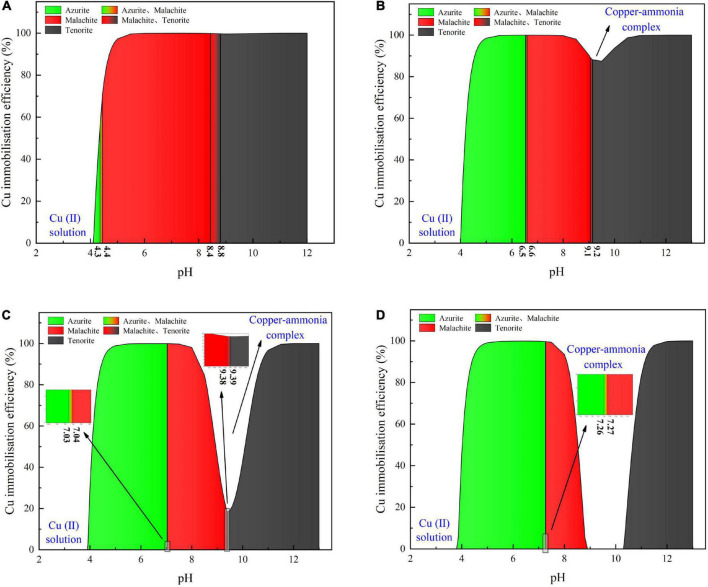
**(A)** copper immobilization efficiency vs. pH relationship for the inoculation proportion = 1:9, **(B)** copper immobilization efficiency vs. pH relationship for the inoculation proportion = 1:3, **(C)** copper immobilization efficiency vs. pH relationship for the inoculation proportion = 1:1, and **(D)** copper immobilization efficiency vs. pH relationship for the inoculation proportion = 3:1 [Cu(NO_3_)_2_ concentration = 40 mM].

**FIGURE 7:**
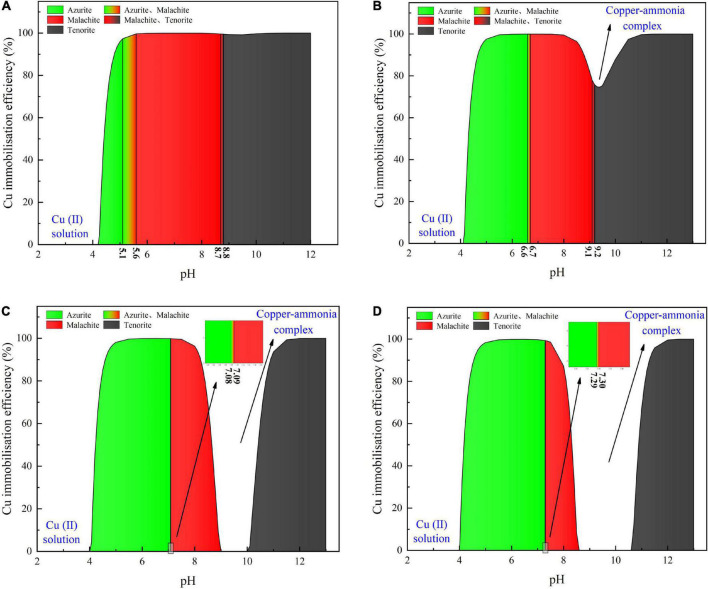
**(A)** copper immobilization efficiency vs. pH relationship for the inoculation proportion = 1:9, **(B)** copper immobilization efficiency vs. pH relationship for the inoculation proportion = 1:3, **(C)** copper immobilization efficiency vs. pH relationship for the inoculation proportion = 1:1, and **(D)** copper immobilization efficiency vs. pH relationship for the inoculation proportion = 3:1 [Cu(NO_3_)_2_ concentration = 20 mM].

**FIGURE 8:**
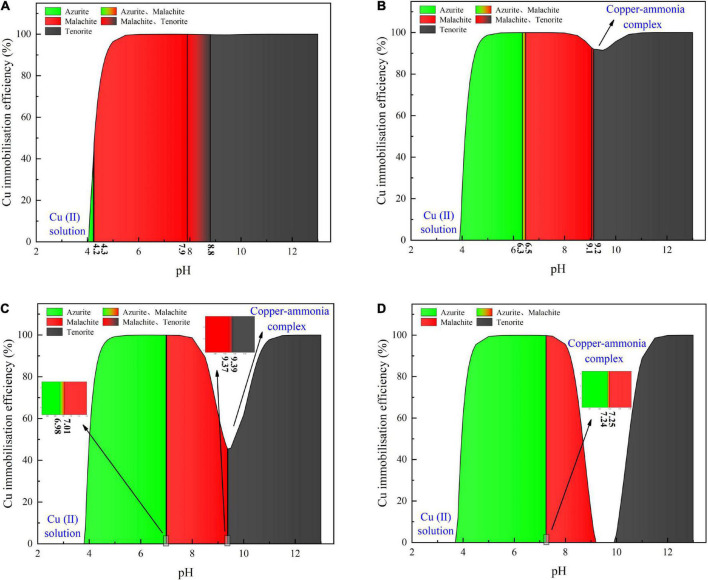
**(A)** copper immobilization efficiency vs. pH relationship for the inoculation proportion = 1:9, **(B)** copper immobilization efficiency vs. pH relationship for the inoculation proportion = 1:3, **(C)** copper immobilization efficiency vs. pH relationship for the inoculation proportion = 1:1, and **(D)** copper immobilization efficiency vs. pH relationship for the inoculation proportion = 3:1 [Cu(NO_3_)_2_ concentration = 60 mM].

Considering harsh pH conditions and the precipitation speciation have been neglected in the test tube experiments, they are interpreted a step further using a series of numerical simulations. Given 40 mM Cu(NO_3_)_2_, the relationships of the copper immobilization efficiency vs. the surrounding pH against the inoculation proportions of 1:9, 1:3, 1:1, and 3:1 are shown in Figure 6. Under the inoculation proportion = 1:9, there are three speciations of carbonate precipitation, including azurite [(Cu_3_(OH)_2_(CO_3_)_2_], malachite [Cu_2_(OH)_2_CO_3_], and tenorite (CuO), when pH remains above 4 (see Figure 6A). The copper immobilization efficiency increases notably when pH is increased from 4.0 to 4.5. It reaches approximately 100% when pH falls within a 5–12 range, with the exception of pH surrounding 9 where a reduction in the copper immobilization efficiency occurs. When pH is below 4, Cu^2+^ are present in a free state and no carbonate precipitation is found, reducing the copper immobilization efficiency to zero. The aforesaid three speciations of carbonate precipitation are also present under the inoculation proportion = 1:3 (see Figure 6B). Similarly, Cu^2+^ are present in a free state when pH remains below 4. Except pH surrounding 9, the copper immobilization efficiency reaches nearly 100% as pH falls within a 5–12 range. Such reduction in the copper immobilization efficiency under the inoculation proportion = 1:1 and 3:1, respectively, is also noted (see Figures 6C,D). It is also present when subjected to 20 mM and 60 mM Cu(NO_3_)_2_, respectively (see Figures 7, 8). In this situation (pH approximately 9) precipitation even disappears under the inoculation proportion = 3:1. Taking a close look at the variations of the copper immobilization efficiency shown in Figures 6–8, higher Cu^2+^ concentrations narrow pH ranges that are associated with the formation of copper-ammonia complexes. For example, pH corresponding to the formation of copper-ammonia complexes is narrowed from 8.2 to 10.2 range when subjected to 20 mM Cu^2+^ to 8.6–10 range when subjected to 60 mM Cu^2+^. In other words, there is a higher possibility for the copper-ammonia complexes to form when subjected to lower Cu^2+^ concentrations. These phenomena are due to the fact that lower Cu^2+^ concentrations in fact turn surrounding environments into alkaline conditions favorable for forming the copper-ammonia complexes. In contrast, higher Cu^2+^ concentrations provide acidic environments.

In acidic environments, the hydrolysis of CO_3_^2–^ is going forward and ammonia is present in NH_4_^+^ form. Considering HCO_3_^–^ and H_2_CO_3_ as well as NH_4_^+^ are not going to react with Cu^2+^, the majority of Cu^2+^ is present in a free state and the remaining is biomineralized with CO_3_^2–^, thereby forming azurite precipitation (see Figures 6–8). Under pH below 4, Cu^2+^ in a free state raises its migration and diffusion potential and is deemed as the main contributor to the reduction in the copper immobilization efficiency. In short, under pH in a 4–6 range (in most cases), the copper immobilization efficiency is attained through azurite precipitation. The copper immobilization efficiency drops sharply to zero under pH below 4 due to Cu^2+^ migration and diffusion. In contrast, the hydrolysis of CO_3_^2–^ is going backward in alkaline environments, and ammonia is, in turn, present in NH_3_ form. NH_3_ is going to react with Cu^2+^, forming the copper-ammonia complexes. The remaining is going to precipitate with CO_3_^2–^ to form malachite precipitation (see Figures 6–8). It is noteworthy that tenorite is precipitated under pH above 10, corresponding to a copper immobilization efficiency of approximately 100%. Notwithstanding that, its chemical and thermodynamic properties are not as good as the other two carbonates (i.e., azurite and malachite) because it dissolves under harsh pH and temperature conditions, causing an inability of preventing Cu^2+^ migration and diffusion. To summarize, under pH surrounding 9, the copper-ammonia complexes notably reduce the copper immobilization efficiency to zero by promoting Cu^2+^ migration and diffusion. In case pH falls within a 7–9 range (in most cases), the copper immobilization efficiency is attained through malachite precipitation.

### Copper immobilization efficiency

**FIGURE 9:**
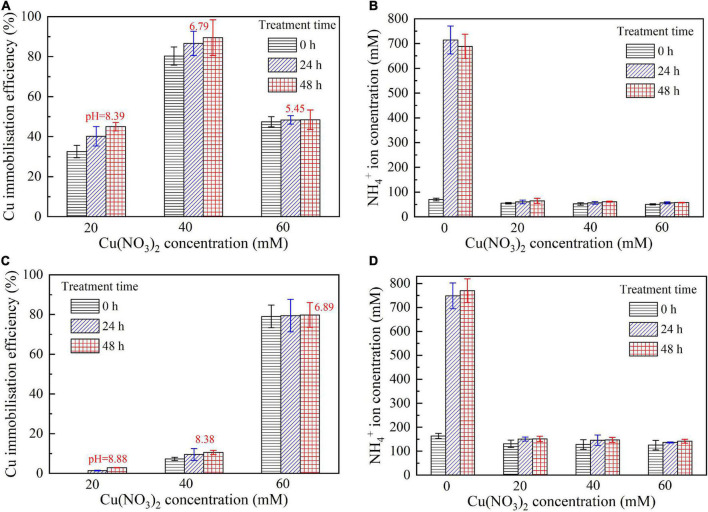
**(A)** copper immobilization efficiency vs. Cu(NO_3_)_2_ concentration relationship and **(B)** NH_4_^+^ concentration vs. Cu(NO_3_)_2_ concentration relationship for the inoculation proportion = 1:9; **(C)** copper immobilization efficiency vs. Cu(NO_3_)_2_ concentration relationship and **(D)** NH4 + concentration vs. Cu(NO_3_)_2_ concentration relationship for the inoculation proportion = 1:3.

**FIGURE 10:**
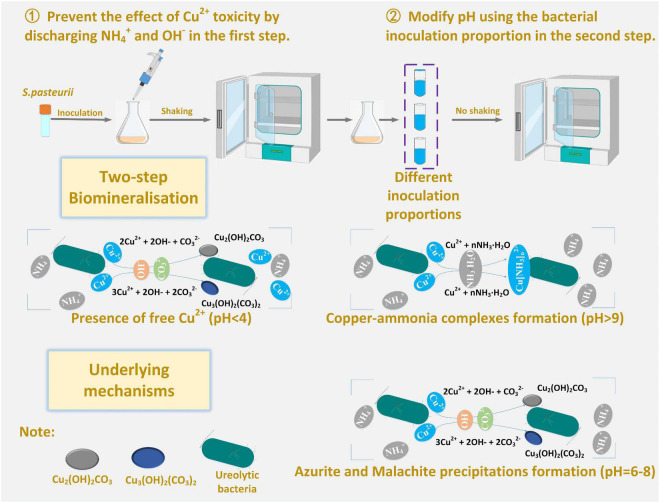
Schematic illustration of the underlying mechanisms affecting the copper immobilization efficiency.

This part aims not only to verify the applicability of the numerical simulations applied to the present work but to investigate further the effect of Cu^2+^ concentration on the immobilization efficiency under a given pH value (Mugwar and Harbottle, 2016). It can be observed that for the inoculation proportion = 1:9, the copper immobilization efficiency being approximately 90% is the highest under Cu(NO_3_)_2_ concentration at 40 mM, corresponding to pH = 6.79 (see Figures 9B,D). Further, the copper immobilization efficiency approximately 45% is the lowest when subjected to Cu(NO_3_)_2_ concentration at 20 mM, which corresponds to pH = 8.39. These results are in line with the simulated results, thereby verifying the applicability of the numerical simulations (see Figures 6–8). The reductions in the copper immobilization efficiencies, when subjected to Cu(NO_3_)_2_ = 20 mM and 60 mM, appear to be attributed to the effect of pH conditions. Although the majority of the ureolytic bacteria lose their activity in the second step of the two-step biomineralization, the discharge of OH^–^ (relevant to bacterial inoculation proportion) in the first step determine not only pH conditions but also the speciation of carbonate precipitation. pH = 8.88, induced by the inoculation proportion = 1:3, gives alkaline environments when subjected to 20 mM Cu(NO_3_)_2_ and promotes the formation of copper-ammonia complexes, yielding the copper immobilization efficiency way below 10% (see Figures 9C,D). In contrast, when subjected to 60 mM Cu(NO_3_)_2_, pH = 6.89, resulting from the inoculation proportion = 1:3, gives acidic environments and then prevent the formation of copper-ammonia complexes, corresponding to the copper immobilization efficiency approximately 80%. In most cases the copper immobilization efficiency using the two-step biomineralization approach higher than 45% is much higher than that using the ordinary biomineralization approach despite a discrepancy in the bacterial inoculation proportion (see Figure 5D). That is to say, the two-step biomineralization approach elevates the copper immobilization efficiency and such improvement is especially pronounced when subjected to higher Cu(NO_3_)_2_ concentrations. However, the copper immobilization efficiency way below 10% under the bacterial inoculation proportion = 1:3 appears when subjected to 20 mM Cu(NO_3_)_2_. Modification concerning pH conditions may consider by reducing the inoculation proportion to 1:9 to prevent the reduction in the copper immobiliztion efficiency. To conclude, the two-step biomineralization approach prevents the effect of Cu^2+^ toxicity by discharging NH_4_^+^ and OH^–^ prior to inoculating the ureolytic bacteria to the liquid medium containing Cu(NO_3_)_2_. Discharging NH_4_^+^ and OH^–^ while cultivating the ureolytic bacteria is deemed as the first step (see Figure 10). To prevent the formation of copper-ammonia complexes, pH conditions are modified by reducing the inoculation proportion, referred to also as the second step. As a result, the copper immobilization efficiency remains very high when even subjected to higher Cu(NO_3_)_2_ concentrations. The use of the two-step biomineralization to secure the urease activity and also to modify pH conditions is considered of great necessity while applying the MICP technology to remedy copper-rich water bodies.

## Conclusion

The proposed two-step biomineralization approach to secure the urease activity and also to modify pH conditions to prevent the copper-ammonia complexes formation was applied to copper immobilization. Based on the results and discussion, some main conclusions can be drawn as follows:

(1) The copper immobilization efficiency way below 10% for the bacterial inoculation proportion = 1:1 is attained and still holds true even when Cu(NO_3_)_2_ concentration is reduced to 5 mM. Although higher inoculation proportions can improve the resistance of the ureolytic bacteria against Cu^2+^ toxicity, their use is accompanied by discharging more OH^–^, turning surrounding environments into alkaline conditions and promoting the formation of copper-ammonia complexes. For this reason, the potential of Cu^2+^ migration is raised, causing an inability of securing the copper immobilization efficiency.
(2) 20–50 mM Cu(NO_3_)_2_ can badly depress the ureolytic bacteria and then reduces the degree of urea hydrolysis. The lower the degree of urea hydrolysis, the lesser the carbonate precipitated, and the lower the copper immobilization efficiency. The lack of CO_3_^2–^, induced by the reduction in the degree of urea hydrolysis, is considered to be the main cause leading to the copper immobilization efficiency way below 10% when subjected to 20–50 mM Cu(NO_3_)_2_.
(3) Under pH in a 4–6 range (in most cases), the copper immobilization efficiency is attained through azurite precipitation. The copper immobilization efficiency drops to zero under pH below 4 due to Cu^2+^ migration and diffusion. Under pH surrounding 9, the copper-ammonia complexes reduce the copper immobilization efficiency to zero. In case pH falls within a 7–9 range (in most cases), the copper immobilization efficiency is attained through malachite precipitation. The findings shed light on the necessity of securing the urease activity and modifying pH conditions while applying the MICP technology to remedy copper-rich water bodies.

## Data availability statement

The original contributions presented in this study are included in the article/supplementary material, further inquiries can be directed to the corresponding author/s.

## Author contributions

Z-FX: data curation, formal analysis, validation, software, and writing—original draft. W-CC: conceptualization, methodology, writing—review and editing, supervision, and funding acquisition. LW: writing—review and editing. Y-XX: writing—review and editing. All authors contributed to the article and approved the submitted version.
